# Endometrial Expression of Insulin Signaling Pathway Genes in Pregnancy Leading to Abortion under 20 Weeks in Infertile Women: A Case-Control Study

**DOI:** 10.22074/IJFS.2021.534736.1163

**Published:** 2022-10-12

**Authors:** Nader Namvarsigaroudi, Zahra Tahmasebi Fard

**Affiliations:** Department of Biology, Roudehen Branch, Islamic Azad University, Roudehen, Iran

**Keywords:** Abortion, Female Infertility, Insulin Receptor, In vitro Fertilization, Unexplained Symptom

## Abstract

**Background:**

Impaired expression of genes which act on hormone signaling pathways is one of the factors affecting miscarriage. In this study, the expression levels of insulin receptor (INSR) and insulin receptor substrates-1 (IRS-1) genes in endometrial tissue of infertile women and fertile women with miscarriage in less than twenty weeks gestation for unknown reasons were evaluated.

**Materials and Methods:**

In this case-control study, forty-two fertile women with children and 42 infertile women, who underwent in vitro fertilization (IVF), were selected. Both groups had abortions under twenty weeks gestation for unknown reasons. The endometrial tissue of all patients was prepared to evaluate the expression of INSR and IRS-1 genes by quantitative real-time polymerase chain reaction (PCR) method after RNA extraction.

**Results:**

There was a statistically significant relationship between the expressions of INSR and IRS-1 genes in the endometrial tissue of the infertile women compared with the fertile women (P=0.002 and P=0.008, respectively). The expression level of genes was decreased in both groups by age and increasing body mass index (BMI). Comparison of genes expression levels in healthy and diabetic participants in each group showed a significant difference (P<0.0001), but no meaningful difference was indicated between diabetic infertile and fertile groups in terms of gene expression. INSR gene expression levels showed an increase in the fertile group in the second 10 weeks and a decrease in IRS-1 gene expression. But in the infertile group, both genes showed a slight increase in expression.

**Conclusion:**

It seems a decreased expression of insulin signaling pathway genes in the endometrial tissue of infertile women can be one of reasons for unspecified abortion. These genes may be strong molecular markers for infertility.

## Introduction

Successful pregnancies in humans and non-human mammals rely on a unique set of events, such as embryo implantation, separation, mating, and parturition. Implantation is associated with molecular and physiological events regulated between the embryo and the receiving endometrium. In the implantation process in humans, fundamental events such as adhesion, adhesion / attachment, invasion, and immune regulation occur (1).

Spontaneous abortion is a significant issue in terms of social and economic effects. Today, most women face the possibility of reduced fertility and increased spontaneous abortion due to delayed pregnancy. Infertility has various causes, the most common of which are tubular and pelvic diseases, ovulation disorders, polycystic ovary syndrome (PCOS) and premature ovarian failure (2).

Insulin is a pivotal metabolic hormone for regulating energy homeostasis in the body. Insulin-dependent signaling also plays an important role in embryo reproduction and early growth (3). In humans, insulin and proinsulin levels (prohormones with less activity than insulin) are significantly associated with weight, height, head circumference, and skin thickness of infants at birth (4). Insulin sends messages through its heterotetrameric receptor. After binding of insulin to alpha extracellular subunits, deformation occurs in the second tyrosine kinase present in the two beta intracellular subunits, resulting in activation of tyrosine kinase to auto-phosphorylate tyrosine components in the Tyr-1158, Tyr-1162, and Tyr-1163 positions, followed by rapid phosphorylation of docking proteins such as insulin receptor substrates (IRS) and several other signaling proteins (5). In endometrial cancer, the insulin hormone, as a growth factor, can increase cell proliferation and inhibit the process of apoptosis through the PI3K/Akt and RAS/MAPK pathways (6, 7). Activation of insulin receptor (*INSR*), insulin receptor substrates -1 (*IRS-1*) and *AKT* has also been linked to the invasive nature of endometrial cancer, and insulin has mitogenic and anti-apoptotic properties for these cells (6).

Human placental growth hormone is increased continuously during the first 20 weeks of gestation, and this hormone has a strong effect on insulin metabolism. Because of this, the insulin signaling pathway is necessary to regulate cell metabolism. In the present study, we hypothesized that energy balance was essential for embryo implantation and growth. Therefore, the disruption of the insulin signaling pathway due to decreased expression of *INSR* and *IR-1* genes in the endometrial tissue of infertile women is considered a factor affecting infertility and abortion in *in vitro* fertilization (IVF). 

## Materials and Methods

### Sample collection

In this case-control study, two groups were selected from the clients referred to the infertility centers of Yas and Mirzakoochak Khan Hospitals in Tehran (2018- 2019). Forty-two women with children, who had experienced at least one normal pregnancy, were selected as the fertile group. Forty-two women without children with a regular menstrual cycle that were married more than one year and also had an unknown reason for infertility were selected as the infertile group. The sample size was calculated based on the following assumption: type1 and 2 errors: 0.05 and 0.20, respectively; expected implantation rate in control group: 65%; expected frequency of abortion: 35%. The infertile group underwent the IVF method to get pregnant, but the fertile group had a normal pregnancy. Both groups had an abortion under twenty weeks for unknown reasons. The aborted fetus also had a normal karyotype. The selection criteria of the groups were as follows: regular menstrual cycles, normal ovarian function, and absence of abnormalities in the uterus and fallopian tubes, or signs of endometriosis on ultra-sonographic or laparoscopic examinations. In addition, the spouses of subjects had sufficient sperm volume; and analysis of semen was according to WHO criteria. Those who did not have this characteristic were excluded.

The subjects ranged in age from 24 to 36 years. Endometrial samples of individuals were collected using a Novak curette/ Pipelle catheter and transferred to a karyotype containing RNA to be stored in liquid nitrogen until RNA extraction. 

### RNA extraction and cDNA synthesis

Approximately 150-200 mg of endometrial tissue samples were washed twice with phosphate buffered saline (PBS, Bioidea, IRAN). Then, the RNA of all samples was extracted with the help of a commercial kit instruction (Invitrogen, Carlsbad, CA, USA). After evaluating the quantity and quality of the extracted RNA according to the kit instructions (Takara Bio Inc., Japan) about 1 mg of the total RNA from each sample was added to random hexamer primers, RT enzyme, and enzyme buffer used for cDNA synthesis and placed in a thermos-cycler.

### Quantitative real-time polymerase chain reaction analysis

Using the ABI StepOne Plus^TM^ system (Applied Biosystems, Germany), gene expression (*INSR* and *IRS-1*) was evaluated by quantitative real time polymerase chain reaction (qRT-PCR). Primers (F: 5′-TTCCGAGACCTCAGTTTCCC-3′ and R: 5′-AGATGACCAGCGCGTAGTTA-3′) were used to proliferate the *INSR* gene, primers (F: 5′-AGGTGGATGACTCTGTGGTG-3′ and R: 5′-GGGATTGTTGAGATGGTGCC-3′) were used for the *IRS-1* gene, and primers (F: 5′-CGTGCGTGACATTAAAGAGAA-3′ and R: 5′-GGGATTGTTGAGATGGTGCC-3′) were used for the *beta-actin* gene (internal control). The proliferation steps included 95°C for 5 minutes for initial DNA denaturation, then 35 cycles at 95°C for 30 seconds, 55°C and 60°C for 30 seconds, and 72°C elongation for 30 seconds. All tests were performed in pairs. Several proliferated products were sequenced. To analyze the sample proliferation, the threshold line was drawn based on the exponential phase of the products to be statistically analyzed using the 2^-ΔΔCT^ method.

### Statistical analysis

Data were analyzed using Graph Pad software version 9. The normal distribution of data was first examined by the Kolmogorov - Smirnov test. Then the variables of age, BMI, duration of marriage and length of pregnancy were calculated based on an independent t test and were reported as mean ± SD. Other data such as diabetes, number of children and abortions were calculated based on Fisher’s exact test between the two groups. The expression level of genes was reported as fold change according to the formula fold change=2^-ΔΔCt^. The fertile and infertile groups were divided into two subgroups for age (30≥ and 30<), body mass index (BMI, 25≥ and 25<), diabetes (healthy and diabetic), length of pregnancy (10 ≥ and 10< week). Fold change of the *INSR* and *IRS-1* gene expression was compared between subgroups, using a two-sample t test. The differences in expression of INSR and IRS-1 genes in the two groups, the effect of age, BMI, diabetes, and length of pregnancy on gene expression were assessed by t-test. The missing data were excluded from the study. In all statistic testes, a P value of less than 0.05 was considered significant. Results were reported with 95% confidence intervals (CIs).

### Ethical considerations

The study protocol conforms to the ethical guidelines of the 1975 Declaration of Helsinki as reflected in a prior approval by the Tehran Islamic Azad University of Medical Sciences (IR.IAU.TMU.REC.1397.007). After obtaining informed consent, the structured questionnaires were filled out by subjects.

## Results

There was no significant difference between the mean age of the two groups, duration of the marriage, number of abortions, smoking and diabetes. In terms of mean BMI, duration of pregnancy and the number of children the groups were statistically significant. Individual information is presented separately in Table 1.

### Evaluation of changes in *INSR* and *IRS-1* gene expression in endometrial tissue of infertile women compared to fertile women

**Fig 1:**
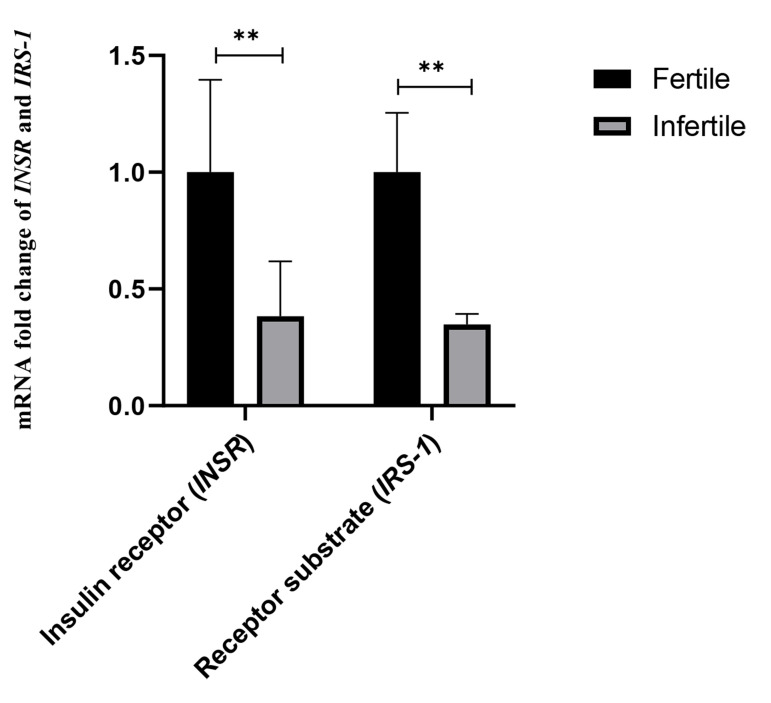
Quantitative real-time polymerase chain reaction (PCR) validation of transcriptome data for *INSR* and *IRS-1* genes. The mRNA fold change was used for comparative gene expression between fertile and infertile women. Independent samples student’s t test was performed to compare *INSR* and *IRS-1* expression between fertile and infertile women.^ **^; P<0.001.

In the fertile group, the expression of the *INSR* gene was 2.61 times higher (P=0.002, 95% CI: 0.639-2.622) and the *IRS-1* gene was 2.87 times higher (P=0.008, 95% CI: 0.177-1.137) than the infertile group. These differences were also statistically significant. The results are shown in Figure 1.

**Table 1:** Baseline characteristics of the infertile and fertile women


Variable		Fertile (n=42)	Infertile (n=42)	P value	95% CI

Age (Y)		34.1 ± 5.80	33.76 ± 5.37	0.770	(-3.76-2.03)
BMI (kg/m^2^)		27.3 ± 2.80	24.09 ± 3.86	<0.0001	(-4.71--1.01)
Duration of marriage (Y)		7.67 ± 3.74	6.50 ± 1.33	0.060	(-2.38-0.05)
Chemical pregnancy or duration of pregnancy (days)		86.1 ± 24.46	19.55 ± 9.93	<0.0001	(49.92-69.65)
Abortion				0.234	(0.47-0.76)
Non		32 (76.19)	36 (85.71)		
1		10 (23.81)	5 (11.90)		
2		0	1 (2.39)		
Diabetes				0.131	(0.15-0.42)
Positive		4 (9.52)	9 (21.43)		
Negative		38 (90.48)	33 (78.57)		
Child				<0.0001	(0.01-0.07)
Non		0	42 (100)		
1		17 (40.48)	0		
2		20 (47.62)	0		
3		5 (11.90)	0		


Data are presented as mean ± SD or n (%). Age, BMI, duration of the marriage and length of pregnancy were calculated based on the independent t test. Fisher’s exact test was used to compare the distribution of other variables (abortion, diabetes and number of children) between the two groups. BMI; Body mass index and CI; Confidence intervals.

### Evaluation of age parameters on the expression of *INSR* and *IRS-1* genes

**Fig 2:**
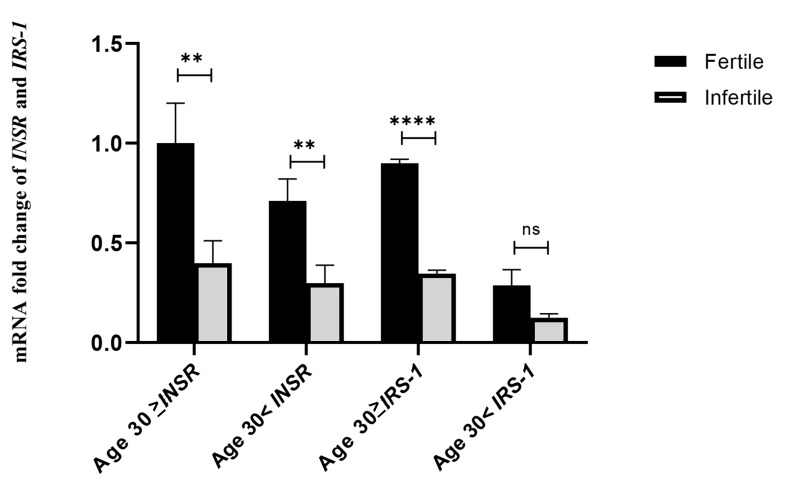
The effect of age parameter on the expression of *INSR* and *IRS-1* genes. The mRNA fold change was used for comparative gene expression between fertile and infertile women. Independent samples student’s t test was performed to compare *INSR* and *IRS-1* expression between the two age groups. ^**^; P<0.001,^ ****^; P<0.00001, and ns; P>0.01.

In terms of age, each group was divided into two subgroups ≤30 and >30 years. Sixteen women in the fertile group and nineteen women in the infertile group were ≤30 years old; and 26 in the fertile group and 23 in the infertile group were >30 years old. In comparison with the fertile group, the expression of the *INSR* gene was 2.95 times (P=0.005, 95% CI: 0.397-4.010) higher and the *IRS-1* gene was 2.92 times (P<0.0001, 95% CI: 0.204-1.719) higher than that of the infertile group with the age of ≤30. The same comparison at age >30 showed that the expression of the *INSR* gene increased by 2.42 times (P=0.001, 95% CI: 0.147-2.459) and the expression of the *IRS-1* gene increased by 1.59 times (P=0.356, 95% CI:-0.131-0.333). Both groups did not differ in the expression of the *IRS-1* gene, except for the ≤30 age range. The results are shown separately in Figure 2.

### Evaluation of the BMI parameter on the expression of *INSR* and *IRS-1* genes

**Fig 3:**
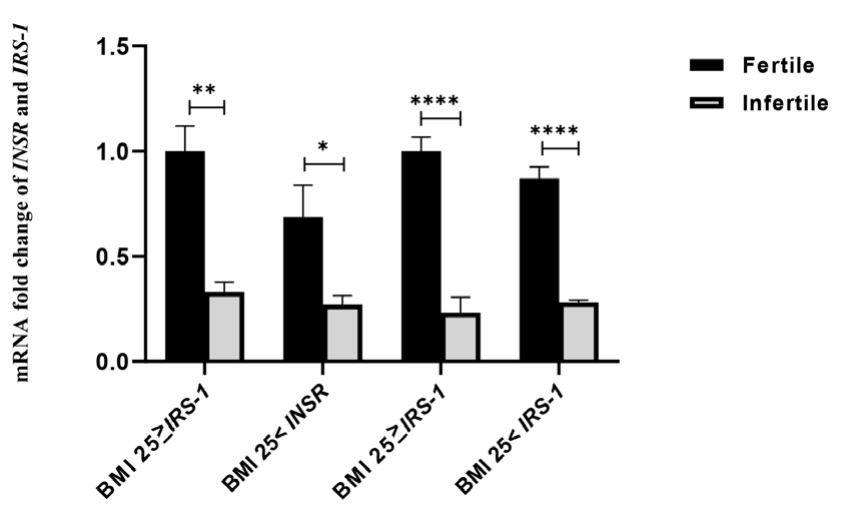
The effect of BMI parameter on the expression of *INSR* and *IRS-1* genes. The mRNA fold change was used for comparative gene expression between fertile and infertile women. Independent samples student’s t test was performed to compare *INSR* and *IRS-1* expression between the BMI of the two groups of women. BMI; Body mass index, ^*^ ; P<0.01, ^**^; P<0.001, and ^****^; P<0.00001.

Twenty-six fertile women and seven infertile ones had a BMI ≤25, and 16 fertile women and 35 infertile ones had a BMI >25. The expression of both genes was decreased by increasing BMI. Comparison of BMI ≤25 in the fertile women compared to the infertile women showed that the expression of the *INSR* gene was 10.07 times (P=0.002, 95% CI: 0.251- 5.161) and *IRS-1* gene was 4.31 times (P<0.0001, 95% CI: 0.533-2.270) higher. Also, fertile and infertile persons at BMI >25 had 1.78 times (P=0.042, 95% CI: 0.214-2.026) more expression of the *INSR* gene and 2.19 times (P<0.0001, 95% CI: 0.069-0.812) more expression of the *IRS-1* gene. A comparison of the subgroups is shown in Figure 3.

### Evaluation of diabetes on the expression of *INSR* and *IRS-1* genes

**Fig 4:**
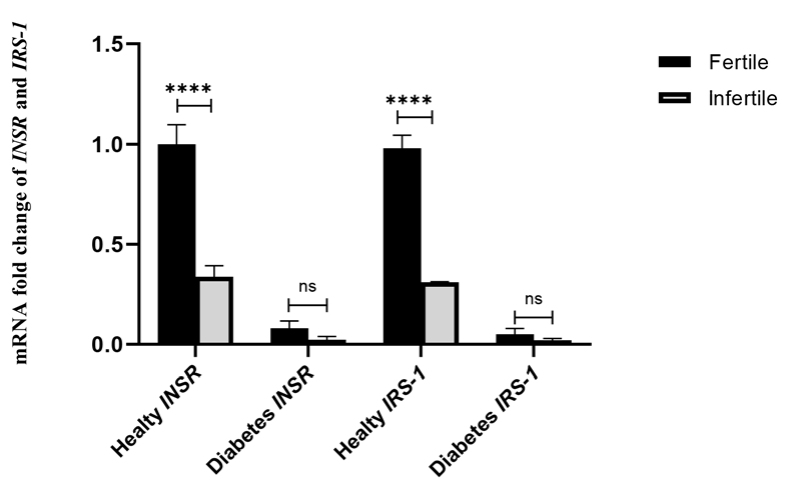
The effect of diabetes on the expression of *INSR* and *IRS-1* genes. The mRNA fold change was used for comparative gene expression between fertile and infertile women. Independent samples student’s t test was performed to compare *INSR* and *IRS-1* expression between women who were healthy or with diabetic disease. ^****^; P<0.00001 and ns; P>0.01.

Nine fertile women and four infertile ones had diabetes (type II). Diabetes affected the expression of genes and caused a reduction in the expression of both genes in subjects with diabetes compared to healthy ones. This difference was statistically significant for the expression of the *INSR* gene (P=0.007) and *IRS-1* gene (P=0.029). Healthy fertile subjects had 23.82 times higher expression of the *INSR* gene than the fertile ones with diabetes (P<0.0001, 95% CI: 2.207-4.293) and 13.83 times higher *IRS-1* gene (P<0.0001, 95% CI: 0.679-1.813). Healthy infertile subjects showed 21.35 times more expression of the *INSR* gene (P<0.0001, 95% CI: 0.230-0.604) than the infertile ones with diabetes and 16.82 times more expression for the *IRS-1* gene (P<0.0001, 95% CI: 0.091-0.152). The comparison of the subgroups is shown in Figure 4.

### Evaluation of the duration of pregnancy on the expression of the *INSR* & *IRS-1* genes

**Fig 5:**
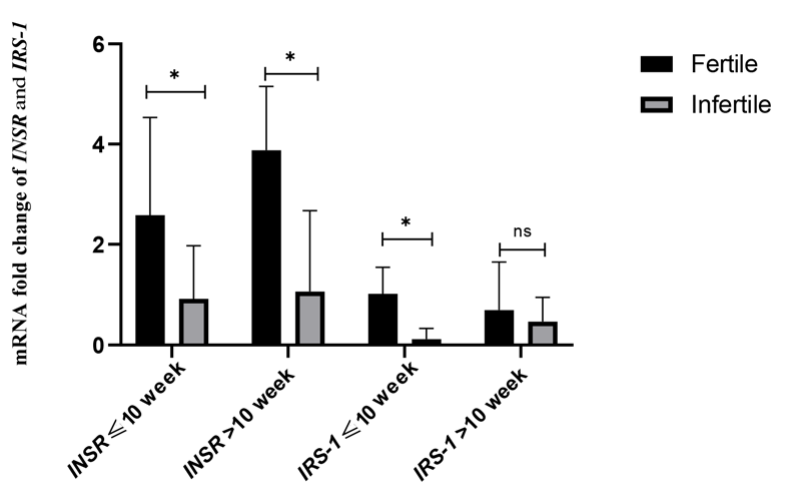
The effect of the length of pregnancy on the expression of *INSR* and *IRS-1* genes. The mRNA fold change was used for comparative gene expression between fertile and infertile women. Independent samples student’s t test was performed to compare *INSR* and *IRS-1* expression and the length of pregnancy between groups. ^*^ ; P<0.01 and ns; P>0.01.

The length of pregnancy was shorter in the infertile group than in the fertile group. This length was divided into two subgroups: ≥10 weeks and <10 weeks. In the fertile group, the expression of the *INSR* gene was 2.79 times (P<0.0001, 95% CI: 0.130-2.503) higher in the first ten weeks of pregnancy and 3.63 times (P<0.0001, 95% CI: 0.697-3.071) higher in the second ten weeks than the infertile group. In terms of *IRS-1* gene expression, the fertile group had 8.71 times (P<0.0001, 95% CI: 0.332- 3.165) more expression in the first ten weeks and 1.48 times (P=0.653, 95% CI:-1.064-0.321) more in the second ten weeks. The results of gene expression in the first ten weeks and the second ten weeks are shown in Figure 5.

## Discussion

Reproduction is controlled by the common function of several neuronal and hormonal signals (neurohormonal system). For central reproduction controlling, the deca-peptide gonadotrophin-releasing hormone (GnRH) is formed to activate the lower elements of the hypothalamus-pituitary-gonadal (HPG) axis, especially the secretion of the famous gonadotrophins luteinizing hormone (LH) and follicle-stimulating hormone (FSH). Also, environmental hormones affect GnRH activity. These gonadal hormones and various metabolic factors are essential for regulating energy homeostasis and fertility. Among these, insulin is a pivotal regulator of the HPG axis. Removing insulin receptors in animal models led to the development of severe metabolic disorders, hypogonadotropism, hypogonadism, and infertility (8). A study by Anjali and his colleagues demonstrated the effect of FSH on the expression of genes related to energy homeostasis. They showed that FSH could increase the expression of the *IRS-2* gene and the functional deficiency of FSH reduced follicular growth and metabolism and led to infertility. 

The pivotal role of the reproductive function of insulin activity in humans is determined by the expression of the insulin receptor in most tissues of the body, the hypothalamus, pituitary, uterus and ovaries (8). The binding of insulin to its INSR receptor causes induction of tyrosine phosphorylation in the insulin receptor substrate (IRS). Then the signal is transmitted through downstream enzymes such as *PI3K* and *AKT2*. Knockout mouse model of INSR causes hyperinsulinemia and hyperglycemia rapidly following diabetic ketoacidosis (9).

Human implantation is a complex and multifactorial process. Successful implantation requires some factors such as a healthy embryo, a receptive endometrium, the molecular coordination between them, and the protection of the host's immunity. Endometrial tissue has a transient functional state and allows blastocysts to be implanted and pregnancy to occur (10). Recent advances in the study of implantation processes have indicated that endometrial acceptance evaluation and pre-implantation genetic testing are necessary to overcome the possibility of implant failure (11, 12) and successful initiation of pregnancy. Early detection of endometrial abnormalities and the discovery of new strategies increase the chances of pregnancy, especially in infertile women.

In this study, a comparison between infertile women who had undergone IVF and fertile women was made. Both groups had an abortion less than twenty weeks for unknown reasons. The infertile group had lower expression of the *INSR* and *IRS-1* genes in uterine tissue compared to the fertile group. This difference of expression was statistically significant. The effect of some variables on gene expression was also evaluated.

Those in each group had less gene expression with aging (over 30 years). This reduction was more in the fertile group than in the infertile group. Comparing infertile with fertile women indicated a significant relationship between aging and the rate of decreased expression of insulin messaging genes. Also, Dunson et al. (13) examined the relationship between age and fertility. Their results demonstrated that women aged 19-26 were significantly more likely to become pregnant than women aged 27-29, and the infertility percent was estimated at 8% for women aged 19-26 and 13 to 14% for women aged 27 to 34, and 18 % for women aged 35 to 39.

The role of obesity is pivotal due to the increased production of hormones derived from adipose tissue, especially leptin (14). Leptin plays a role in energy balance and reproduction (8). Lack of leptin signaling in rats and humans causes obesity and infertility. Increased leptin in obese people reduces the activity of the hypothalamic-pituitary- gonadal (HPG) axis by creating a state of resistance (14). In the current study, the subgroups with BMI ≥25 and BMI <25 were also examined. Thirty infertile individuals and only sixteen fertile individuals had a BMI >25. Comparison of the two groups showed that the expression of both genes is decreased by increasing BMI. In obese fertile women, expression of both genes decreased significantly, but the infertile group showed a slight expression decrease in the *INSR* gene and an increased expression in the *IRS-1* gene.

Because insulin directly stimulates GnRH secretory activity (8), hyperglycemia occurs by decreased insulin secretion in diabetes. Also, diminished insulin secretion leads to infertility for reasons such as damage to the hypothalamic-pituitary-gonadal axis, increased DNA damage, oxidative stress, increased endoplasmic reticulum stress, mitochondrial function damage, and cell pathway modulation. Regulation of insulin levels directly affects *INSR* and *IGF1R* expression. Also, it leads to activation of signaling pathways associated with cell proliferation, differentiation, metabolism, and survival. In men with unexplained infertility, the lack of *INSR* and *IGR1R* in Sertoli cells causes reduction of testicular size by 75% and daily sperm production (15), insulin resistance also affects reproductive anomalies and their metabolism (16). In the present study, women with diabetes in both groups had a low-level expression of *INSR* and *IRS-1* genes compared to healthy subjects. But comparing infertile women with diabetes with fertile women with diabetes did not indicate a significant difference in terms of gene expression.

Concerning the length of pregnancy until termination, the two groups were divided into two subgroups of women less than 10 weeks pregnant and the women in the second 10 weeks of pregnancy. It aimed at evaluating the expression levels of *INSR* and *IRS-1* genes. The fertile women in the second 10 weeks of pregnancy showed that the expression levels of *INSR* and *IRS-1* genes increase and decrease, respectively. Infertile women in the second 10 weeks had a slight increase in *INSR* gene expression compared to the women in the first 10 weeks. They had a significant increase in *IRS-1* gene expression. It seems that decreasing or increasing one of the genes could disrupt the insulin signaling pathway.

## Conclusion

Hormones affect fertility and cause changes in gene expression for implantation and fetal growth through messaging pathways. Disorders in the signaling pathway of endometrial tissue can be one of the reasons for the lack of fetal growth and abortion. One of the most important hormones is insulin, which transmits the message inside the cell through the receptor and the receptor substrate. Genetic changes in infertile women lead to reduced expression of these proteins and disrupted hormone signaling. Other factors such as obesity, diabetes, old age and smoking also reduce the expression of these genes and aggravate the problem of infertility. Therefore, it is apparent that genetic disorders are one of the factors affecting infertility.
